# Development of Antimicrobial Coatings by Incorporating Curcumin and Silver-Based Additive into a Commercial Water-Based Paint

**DOI:** 10.3390/biomimetics11090659

**Published:** 2026-09-12

**Authors:** Francesca Pescosolido, Silvia Vesco, Felicia Carotenuto, Andrea Ciammaruconi, Florigio Lista, Roberto Bei, Paolo Di Nardo, Federica Trovalusci

**Affiliations:** 1 Department of Clinical Sciences and Translational Medicine, University of Rome “Tor Vergata”, 00133 Rome, Italybei@med.uniroma2.it (R.B.); 2 CNR-IMM, Institute for Microelectronics and Microsystems IMM, 00133 Rome, Italy; 3 Department of Enterprise Engineering “Mario Lucertini”, University of Rome “Tor Vergata”, 00133 Rome, Italy; 4 Interdepartmental Center for Regenerative Medicine (CIMER), University of Rome “Tor Vergata”, 00133 Rome, Italy; 5 Defence Institute for Biomedical Sciences, Via Santo Stefano Rotondo 4, 00184 Rome, Italy; andrea.ciammaruconi@gmail.com (A.C.);

**Keywords:** curcumin, silver ions, antibacterial surfaces, antimicrobial coatings

## Abstract

Antibacterial coatings are essential for preventing infections and maintaining adequate hygiene standards in high-density and high-risk environments, such as public spaces, public transportation systems, schools, and healthcare facilities. In this context, it is essential that such coatings are easy to apply and compatible with large-scale production processes. Among the various strategies for developing antimicrobial surfaces, the incorporation of antibacterial agents into paints represents a particularly practical and versatile approach. In this work, antimicrobial coatings were developed by incorporating curcumin, a naturally derived antibacterial compound, into a commercially available water-based paint. For comparison, coatings were also developed by incorporating a commercially available silver-ion-based additive, a well-established antibacterial agent, into the same paint. Antimicrobial dispersions were deposited onto polycarbonate (PC) and polymethyl methacrylate (PMMA) substrates using a spray coating technique. The produced coatings were evaluated in terms of adhesion and hardness according to the ASTM D3359 and ASTM D3363 standards, respectively, achieving ratings of 4B for adhesion and 4B/5B for hardness. Moreover, after five days of continuous water immersion, no visible signs of cracking, delamination, or discoloration were observed. The resulting curcumin- and silver-based coatings, under the tested surface-contact assay conditions, markedly reduced viable bacterial recovery against both *S. aureus* and *E. coli*, with no colonies recovered at the dilution range used for comparison with the Paint-Blank control. These results support the potential of curcumin as a naturally derived antibacterial additive for the development of water-based antibacterial coatings and provide a basis for further investigation of their long-term stability, durability, and practical applicability.

## 1. Introduction

Although direct person-to-person contact, through saliva droplets, hand-to-hand transmission, or close proximity, remains the primary route for the spread of bacterial infections, contaminated surfaces can also facilitate transmission and pose a significant risk to human health [1,2,3,4]. In particular, when contaminated surfaces are present in hospital environments, such as walls, counters, computers, medical records, desks, or beds, they can significantly contribute to the spread of infections among patients and lead to serious complications [2,5,6,7,8]. This issue, in combination with the emergence of antibiotic-resistant bacterial strains [9,10,11], has prompted scientific research to develop new strategies to prevent bacterial proliferation on surfaces [12,13]. The most common approach involves the design of surface coatings with bactericidal activity. Antibacterial coatings are typically classified into three categories based on their killing mechanisms: (i) bacteria-repellent surface; (ii) contact-killing surface; and (iii) antibacterial-releasing surface [14,15,16].

Given their widespread use, paints represent an attractive platform for the development of antibacterial coatings. Paints are extensively applied to a broad range of surfaces, including the exterior facades of urban structures and the interiors of residential, educational, commercial, and healthcare buildings, as well as the furnishings and spaces within these environments [17].

Paint formulations are highly complex, typically consisting of polymers, pigments, thinners, and various additives [18] that modulate properties such as viscosity, applicability, drying time and adhesion to different substrates. These formulations are tailored to meet specific performance requirements and continue to evolve in response to market demands.

In recent years, increasing awareness of the environmental impact of human activities has driven the need to develop more sustainable paint formulations. In particular, efforts have focused on reducing the use of volatile organic solvents. The industry has progressively shifted toward the production of water-based paints [17,19,20].

In this context, commercial water-based paints offer a practical and potentially more environmentally favorable matrix for the development of antibacterial coatings, as they are readily available and easily applicable to diverse substrates.

For the development of antimicrobial coatings, paints can be modified by incorporating chemical additives with specific biocidal activities [3,17,21].

This approach enables the production of coating systems capable of inhibiting bacterial growth and suitable for application to a wide range of surfaces, including wood, walls, plastics, and glass.

A wide range of inorganic and organic chemical compounds have been studied and used as antibacterial additives. These include metals, polymers and natural compounds [15,22,23,24,25]. In particular, the antimicrobial activity of metallic nanoparticles, such as silver, is well documented in the scientific literature [26,27,28,29]. In recent years, increasing attention has been devoted to antimicrobial coatings based on naturally derived bioactive compounds. These approaches exploit plant-derived naturally occurring molecules with intrinsic antibacterial activity for the development of sustainable functional coatings [30,31].

Among these, curcumin has emerged as a promising candidate. Curcumin is a naturally occurring polyphenolic compound extracted from *Curcuma longa* [32,33]. It is considered a secondary metabolite of plant origin, whose biosynthesis can be influenced by physiological and environmental stimuli [34,35]. Curcumin exhibits a range of intrinsic bioactive properties, including antioxidant, anti-inflammatory, and antibacterial activities, which make it particularly attractive for biomedical applications [33,36,37]. In this context, curcumin has attracted significant interest as a biofunctional additive for the development of functional materials, owing to its intrinsic bioactivity and potential to impart specific biological properties to the resulting materials.

The inhibitory effect of curcumin on the growth of both Gram-positive and Gram-negative bacteria, including *Staphylococcus aureus* (*S. aureus*), *Escherichia coli* (*E. coli*), *Enterococcus faecalis* (*E. faecalis*), and *Pseudomonas aeruginosa* (*P. aeruginosa*), is well documented in the literature [37,38,39].

Accordingly, numerous studies have investigated the use of curcumin in coating systems based on different matrices and deposition approaches for the development of functional and antimicrobial systems [40,41].

For example, Ibrahim et al. [42] reported a chitosan–curcumin coating for Mg alloy substrates, which exhibited both antibacterial and anticorrosive potential. Oves et al. [43] developed an antibacterial coating based on curcumin nanoparticles combined with graphene for application on cotton and other textile substrates, demonstrating significant antibacterial efficacy. Sharma et al. [44] developed an antibacterial ink for food-packaging applications, formulated by combining curcumin with an aqueous acrylic resin. The resulting formulation was applied as a coating onto polyethylene terephthalate (PET) substrates.

Among the various studies reported in the literature, Querido et al. [30] investigated the incorporation of curcumin into a commercial paint, providing a comprehensive assessment not only of its antibacterial activity but also of the potential toxicity of the resulting coatings.

In our previous work [45], the feasibility of incorporating curcumin into a commercially available water-based paint was demonstrated, and the formulation and spray coating deposition were established. However, the antimicrobial performance and functional properties of the resulting coatings were not investigated.

Building upon these preliminary findings, the present work aims to assess the antibacterial activity and key functional properties of the developed curcumin-based system, comparing them with those of a corresponding coating prepared using a commercially available silver-ion-based additive. Paint dispersions were produced by separately incorporating curcumin and the silver-ion-based additive into a commercial water-based paint. In particular, the modified paints were applied by spray coating onto polycarbonate (PC) and polymethyl methacrylate (PMMA) substrates, two widely used polymeric materials with potential relevance to applications involving surfaces in public and healthcare environments [46,47]. The resulting coatings were characterized in terms of their structural properties, adhesion, hardness, and water stability. Their antibacterial activity was evaluated against representative Gram-positive and Gram-negative bacterial strains, *S. aureus* and *E. coli*. The silver-ion-based additive was selected as a reference formulation to compare the antibacterial performance and key coating properties of the curcumin-based system with those obtained using an additive with well-established antibacterial activity and widespread use in industrial and commercial applications.

This work therefore builds upon previous studies investigating the use of curcumin as an antibacterial additive and further assesses its potential for the development of antibacterial coatings by combining the evaluation of antibacterial activity with the characterization of key coating properties relevant to practical application, including adhesion, hardness, and water stability.

## 2. Materials and Methods

### 2.1. Reagents

Curcumin was acquired from Acros Organics Chemicals (Thermo Fisher Scientific, Waltham, MA, USA); ethanol was purchased from Merck. Silver ions supported on ultraporous nanosized glass spheres were purchased from Sanitized (Sanitized, Burgdorf, Switzerland) and are hereafter referred to as Ag^+^. Acrimax water-based white commercial paint (MaxMeyer, Milan, Italy) was selected as the matrix for the dispersion of the biocidal agents. The commercial paint was experimentally modified by incorporating curcumin or Ag^+^, followed by dilution with water.

Substrates: PC 50 mm × 50 mm and PMMA 50 mm × 50 mm. Prior to use, the substrates were cleaned with 99% ethanol.

The strain *E. coli* ATCC 8739 was obtained from Thermo Scientific (Waltham, MA, USA), while *S. aureus* ATCC 6538P was obtained from Microbiologics (St. Cloud, MN, USA), and both are recommended for antimicrobial susceptibility testing.

### 2.2. Development of Antimicrobial Dispersions

The antimicrobial dispersions are produced following the methodology previously outlined in [45]. Specifically, the dispersion preparation includes the following steps:

- Appropriate amounts of curcumin powder were weighed and directly added to the commercial paint to obtain final curcumin concentrations of 0.1, 0.5, and 1 wt%, calculated with respect to the initial mass of the paint.
- The resulting Paint–Curcumin dispersions were ultrasonicated for 1 h to promote the homogeneous dispersion of curcumin within the paint matrix.
- Subsequently, distilled water was added at 10 wt%. The resulting dispersions were then subjected to an additional 30 min of ultrasonication to further promote homogenization prior to application onto the PC and PMMA substrates.

Paint-Ag antimicrobial dispersions were produced following the same procedure and under the same experimental conditions, with Ag^+^ used as the antimicrobial additive instead of curcumin.

Additionally, Paint-Blank reference formulations were prepared by diluting the paint with distilled water to obtain a final water content of approximately 10 wt%.

The dispersion names and their corresponding additive concentrations are reported in Table 1.

### 2.3. Coating Methodology

Antimicrobial dispersions are applied on PC and PMMA substrates using the spray coating methodology as described in [45]. The HVLP spray gun equipped with a 1.5 mm nozzle is used to spray the dispersions at a gun pressure of 2.5 bar.

The spray gun was moved horizontally across the substrate surface; at least four overlapping spray passes were applied to ensure complete and uniform coverage of the substrate surface. The dispersions containing 0.1, 0.5, and 1 wt% antimicrobial agents were spray-coated onto PC substrates. As reported in Table 1, the 1 wt% formulations were also deposited onto PMMA substrates to evaluate the influence of the substrate type on coating performance. After deposition, the coated specimens were allowed to dry under ambient laboratory conditions for 10 days before characterization and testing.

The coating thickness was determined using a digital micrometer (resolution: 0.001 mm) by measuring the substrate thickness before and after coating deposition at three different positions on each specimen. The coating thickness was calculated as the difference between the corresponding measurements. The average coating thickness ranged from approximately 30 to 60 μm. The mean coating thickness and standard deviation for each coating formulation are reported in the Supporting Information (Tables S1 and S2 and Figure S1).

### 2.4. Antimicrobial Test

***MIC determination of curcumin and Ag+***

The antimicrobial activity of the two additives, curcumin and Ag^+^, was evaluated using the broth microdilution method. For this purpose, curcumin was initially dissolved in acetone at a concentration of 10 mg/mL, while Ag^+^ was resuspended at 10 mg/mL final concentration in water.

For minimum inhibitory concentration (MIC) evaluation, working solutions were prepared by mixing equal volumes of antimicrobial solutions and dimethyl sulfoxide (DMSO). These working solutions were then serially diluted and added to the appropriate wells of microtiter plates containing Mueller-Hinton broth. Experimental controls containing the same final concentrations of acetone and/or DMSO used in the corresponding antimicrobial-agent wells were included to exclude solvent-related growth inhibition. The resulting solutions were used to determine the MIC.

Mueller–Hinton (MH) medium, either in the form of agar (MHA) or broth (MHB) [48] was used for bacterial inoculum preparation. A reference 0.5 McFarland suspension of bacteria was prepared by measuring turbidity in a spectrophotometer, with an absorbance between 0.08 and 0.13 at a wavelength of 625 nm [48]. The obtained inoculum of bacteria to be used in microtiter plate wells was also controlled. To verify the final inoculum concentration, 10 μL was added to 10 mL of broth, and then 100 μL from this dilution was placed and spread onto MHA plates and incubated for 12–18 h at 37 °C. The growth of 20–80 colonies of bacterial strain indicates a density of 5 × 10^5^ CFU/mL.

Bacterial suspensions of *E. coli* (ATCC 8739) and *S. aureus* (ATCC 6538P) were prepared from bacterial strains stored at −80 °C in cryovials containing porous beads for microbiological culture preservation and cultured overnight in liquid medium. The inoculum to be used for MIC evaluation was prepared by comparison with a suspension of 0.5 McFarland. The inoculum was added to the liquid medium with the antimicrobial agent within 10 min after preparation, to maintain a consistent cell density (CFU/mL).

***Plate preparation***

The microorganisms were subjected to a microdilution test in an Eppendorf Cell Culture 96-well Plate (Eppendorf, Hamburg, Germany) to determine the MIC of curcumin and Ag^+^. Antimicrobial suspension (100 µL) of curcumin or Ag^+^ was dispensed into the first well of the column of the microplate, followed by serial twofold dilutions. Finally, 100 µL of bacterial inoculum containing approximately 1  ×  10^5^ CFU/mL of bacteria (*E. coli* or *S. aureus*) was dispensed in each well, resulting in a final concentration of 5  ×  10^4^ CFU/mL and a total volume of 200 µL per well. The final concentration of antimicrobial molecules, after the addition of bacteria, ranged from 5 mg/mL to 0.019 mg/mL. As a positive growth-inhibition control, a column containing ethanol and bacterial suspension was prepared, with EtOH concentrations from 50 wt% to 0.39 wt%. The microplate was covered with a sterile cover and incubated for 18–24 h at 37 °C. Microbial growth was determined by turbidity; the lowest concentration exhibiting absence of visible turbidity was identified as the MIC. Three independent experiments were performed, and each was run in triplicate.

***Antimicrobial activity of the coatings***

The antibacterial activity of the fabricated surfaces was assessed using a modified surface-contact assay adapted from ISO 22196 and performed under high-bacterial-load conditions [49]. This high bacterial load was intentionally selected to challenge the coatings under stringent experimental conditions, considerably exceeding the bacterial burden typically expected on environmental surfaces. The tested bacterial inoculum was adjusted and diluted in Nutrient Broth (Fischer Scientific, Hampton, NH, USA) to obtain the initial concentration of 5–10 × 10^9^ CFU/mL, corresponding to approximately 5 × 10^8^–1 × 10^9^ CFU applied to each coated sample. The bacteria were collected by centrifugation (5 min at 4000 rpm) and resuspended in 1x buffered phosphate saline (PBS). Paint-Blank, Paint-Curcumin(0.1), Paint-Curcumin(0.5), Paint-Curcumin(1), Paint-Ag(0.1), Paint-Ag(0.5), and Paint-Ag(1) coated surfaces were placed in a sterile Petri dish, inoculated with 100 μL of bacterial preparation then covered with the sterile piece of film (polyethylene, dimensions of 40 mm × 40 mm, 0.1 mm thick) to prevent the spread of the inoculum from the edges, keep it in place and avoid dehydration of bacteria. Treated and untreated samples were tested in triplicate. The samples were incubated at 37 °C with a relative humidity of at least 90% for 18 h. At the end of incubation, 10 mL of PBS 1X was added to each sample, placed on a shaker, and mixed thoroughly to facilitate the release of the bacteria from the sample surface. Serial dilutions of the PBS containing the bacteria were plated on Plate Count Agar. For each experiment, serial dilutions were selected to obtain countable colonies from the Paint-Blank control, confirming efficient recovery of viable bacteria from untreated coatings. The same serial dilution and plating procedure was then applied to the antimicrobial-coated samples to allow direct comparison with the Paint-Blank control, and residual viable bacteria were recorded as CFU recovered per sample. All plates were incubated at 35 °C for 24 h. After incubation, bacterial colonies were counted and recorded. For each condition, bacterial reduction was calculated from colony counts obtained at the dilution range used for comparison with the Paint-Blank control and expressed as the percentage decrease in viable recovery relative to this control. When no colonies were recovered from antimicrobial-coated samples at the dilution range used for comparison with the Paint-Blank control, viable recovery was considered below the quantification range of the assay. All antibacterial coating assays were performed in three independent replicates.

### 2.5. Characterization Techniques

Chemical and mechanical characterizations were performed on the Paint-Blank, Paint-Curcumin(1), and Paint-Ag(1) coatings applied onto both PC and PMMA substrates to assess the effect of antimicrobial additive incorporation at the highest concentration investigated, representing the most challenging condition for the coating system.

FT-IR spectra of the coatings were recorded in absorbance mode using a Thermo Scientific Nicolet Summit spectrometer with a diamond ATR accessory. Spectra were collected over 4000–400 cm^−1^ at 2 cm^−1^ resolution.

Coating adhesion to the substrates was evaluated using the cross-cut tape test in accordance with ASTM D3359. A cross-hatch pattern was produced on the coated surface using a multi-blade cutter. Adhesive tape was then applied firmly over the incised area, maintained in contact for approximately 1 min, and removed by pulling it back at a consistent angle. The adhesion rating was determined from the amount of coating detached after tape removal and classified according to the ASTM D3359 rating scale as follows: 5B (0% coating removal), 4B (<5%), 3B (5–15%), 2B (15–35%), 1B (35–65%), and 0B (>65% coating removal [50,51,52].

The coating hardness was evaluated using the pencil hardness test according to ASTM D3363. Pencils from 6B (softest) to 6H (hardest) were sharpened before each test and placed on a Byk tool holder at an angle of 45° and fixed by a screw [52,53,54]. The coated samples were placed on a horizontal surface, and the holder was manually pushed across the sample surface to evaluate the resistance of the coating to scratching.

To evaluate the potential application of these dispersions as coating agents for inert surfaces, such as countertops and desktops, their stability under water exposure was investigated, as these surfaces are routinely subjected to cleaning with aqueous solutions. Water resistance was assessed by partially immersing the coated samples in 50 mL of deionized water for five days. The coated specimens were masked with Kapton^®^ tape to protect the lateral surfaces and define the immersed area, which was approximately 5 × 1.5 cm^2^. The immersed area was defined by the water level, with 1.5 cm corresponding to the height of the substrate covered by water and 5 cm corresponding to the substrate base. The beakers were sealed with Parafilm^®^ to minimize water evaporation throughout the test.

After the immersion period, the coatings were examined using a digital microscope (Hirox HR-2016, Hirox Co., Ltd., Tokyo, Japan) at 130× magnification. The coatings were visually inspected for evidence of degradation, including delamination, crack formation, or coating removal, by comparing the immersed and non-immersed regions. The tests were conducted on two specimens for each substrate type.

## 3. Results and Discussion

### 3.1. Visual Appearance of the Coatings and Their Antibacterial Activity Evaluation

**Figure 1:**
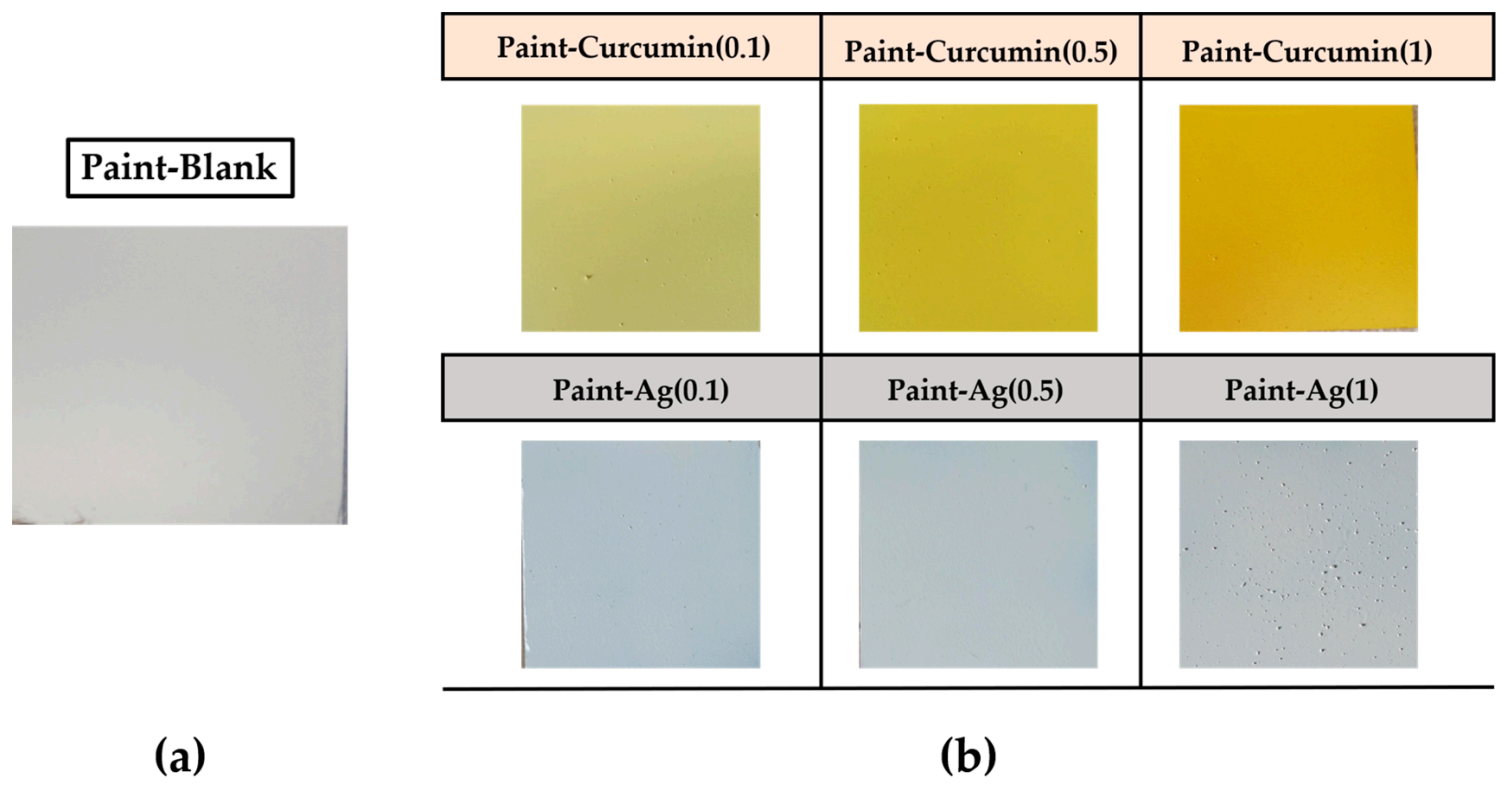
Pictures of (**a**) Paint-blank; (**b**) Paint-curcumin (**top**) and Paint-Ag coatings (**bottom**) on PC substrates.

Figure 1 displays the appearance of the substrates following paint application.

As shown in Figure 1a, the Paint-Blank coating exhibits a white, opaque appearance. The incorporation of curcumin (Figure 1b, top) imparts a yellow–orange coloration to the surfaces, consistent with the characteristic hue of curcumin [55]. The Paint-Curcumin(0.1) coating exhibits a less intense coloration compared to the coatings containing higher percentages of curcumin (Paint-Curcumin(0.5) and Paint-Curcumin(1)), indicating that the color intensity increases with increasing curcumin content.

Instead, the inclusion of Ag^+^ (Figure 1b, bottom) does not alter the original color of the paint. However, at the highest Ag^+^ content, corresponding to the Paint-Ag(1) coating, the presence of agglomerates is observed on the surface, likely indicating a less homogeneous dispersion.

The antibacterial activity of the coatings was evaluated to assess the antibacterial performance of the resulting surfaces.

Prior to coatings evaluation, the antibacterial activity of the free additives, curcumin and Ag^+^, was assessed by determining their MICs against *E. coli* and *S. aureus*. The resulting MIC values are reported in Table 2.

As shown in Table 2, curcumin exhibited a MIC of 0.15 mg/mL against *S. aureus*, while against *E. coli* it showed a MIC of 1.25 mg/mL. These values are in line with those reported in the literature [56,57,58] and suggest a species-dependent antibacterial activity of curcumin, with lower susceptibility observed for *E. coli* compared with *S. aureus*, in agreement with previous reports on the variable antibacterial efficacy of curcumin [56,57].

In contrast, Ag^+^ effectively suppressed the growth of both bacterial strains, exhibiting an MIC of 0.62 mg/mL, a value in line with those reported in the literature [59,61]. As reported in Table 2, however, the MIC values for silver vary considerably depending on the nature and form of the silver species. Indeed, silver has been investigated in different forms, including nanoparticles [60,61], and silver incorporated into porous structures, such as zeolites, which can release ionic and/or metallic silver species [59,62]. The physicochemical characteristics of the silver species, including their oxidation state, aggregation state, particle size, and degree of ion release, can affect their interaction with bacterial cells and, consequently, the resulting MIC values [59,63]. In the present study, the Ag^+^ are supported on ultraporous nanosized glass spheres and were therefore not present as free ions in solution. Despite this difference in formulation and silver availability compared with some of the systems reported in the literature, the relatively low MIC obtained in this work is consistent with the antibacterial activity reported for other silver-based systems. These results indicate that the supported Ag^+^ species retain a significant antibacterial effect, despite being immobilized on the glass substrate.

The antibacterial activity of all produced coated surfaces listed in Table 1 was evaluated against *E. coli* and *S. aureus*. Considering the inoculum concentration and the 100 µL applied to each surface, each coated sample was challenged with approximately 5 × 10^8^–1 × 10^9^ CFU. After 18 h of contact, viable bacterial recovery from the Paint-Blank coating was clearly detectable and countable, providing the reference control used to calculate bacterial reduction. Conversely, no colonies were recovered from any curcumin or Ag^+^ coating for either bacterial strain at the same dilution range used for comparison with the Paint-Blank control. Under these dilution conditions, this was reported as 100% bacterial reduction relative to the Paint-Blank control. The results are summarized in Table 3.

As reported in Table 3, no concentration-dependent differences in antibacterial performance were observed, as no viable colonies were recovered from any curcumin- and Ag^+^-containing coatings at the dilution range used for comparison with the Paint-Blank control. This suggests that, after 18 h of contact with the coated surfaces, even the lowest antimicrobial additive concentration investigated was sufficient to reduce viable bacterial recovery below the quantification range of the assay.

**Figure 2:**
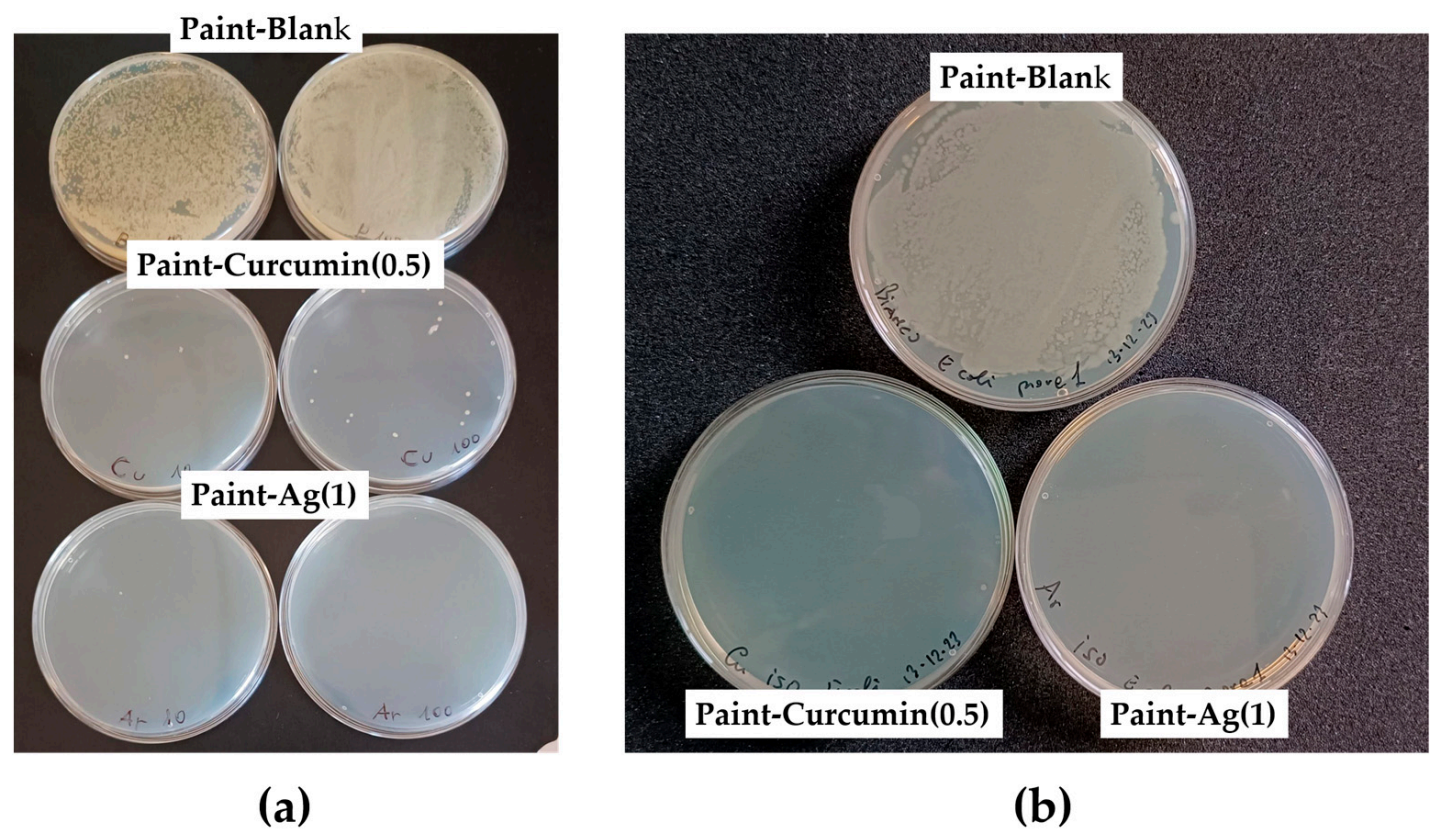
Representative agar plates showing viable bacterial recovery from undiluted recovery suspensions after 18 h of contact with (**a**) *S. aureus* and (**b**) *E. coli*. The formulations shown are Paint-Blank, Paint-Curcumin(0.5), and Paint-Ag(1.0).

Figure 2 shows representative agar plates obtained by plating undiluted recovery suspensions from selected samples, namely Paint-Blank, Paint-Curcumin(0.5), and Paint-Ag(1). This undiluted-plating approach was used for visual comparison and was not intended for quantitative enumeration of the Paint-Blank control. Quantitative bacterial reduction values were instead calculated from serial dilutions selected to obtain countable colonies from the Paint-Blank control, as reported in Table 3. Overall, the developed coatings markedly reduced viable bacterial recovery of both *E. coli* and *S. aureus* under the tested surface-contact conditions. These findings are consistent with previous studies reporting antibacterial activity of curcumin-based [42,43,64,65] and silver-based coatings [66,67,68]. A comparison with selected studies [30,42,65,66,67,68,69] on antimicrobial coatings containing curcumin, silver, or other naturally derived materials is provided in Table S3.

A relevant comparison can be drawn with the study by Querido et al. [30], who incorporated curcumin at a concentration of 0.4 mg/mL into a commercial acrylic paint and evaluated its antibacterial performance using a surface-contact method based on ISO 22196/JIS Z 2801 against five bacterial strains. Since differences exist between their study and the present work in terms of the specific experimental protocols, paint formulations, and curcumin concentrations investigated, only a qualitative comparison can be made. Querido et al. reported that the curcumin-containing paint reduced viable bacterial counts compared with the unmodified paint. The antibacterial effect was most pronounced against Gram-positive bacteria (*S. aureus*), then Gram-negative (*E. coli*). The authors suggested that the relatively limited antibacterial effect, particularly against Gram-negative bacteria, may be related to the curcumin concentration used (0.4 mg/mL), which was below than several MIC values reported in the literature, as shown in Table 2.

In the present study, all three curcumin concentrations investigated resulted in a marked reduction in viable bacterial recovery for both *E. coli* and *S. aureus* under the tested surface-contact conditions. Notably, even the lowest curcumin concentration (Paint-Curcumin(0.1)) investigated was sufficient to reduce viable bacterial recovery below the quantification range of the assay. Although the results cannot be directly compared quantitatively, the more pronounced antibacterial response observed in the present study may be related, at least in part, to the different curcumin concentrations investigated, while differences in paint formulation and experimental protocol may also have contributed to the observed results. Overall, the obtained results further support the potential of curcumin as an antibacterial additive for the development of antibacterial paint.

However, it should be noted that free curcumin showed lower activity against *E. coli* than against *S. aureus* in the microdilution test. This finding is consistent with the strain-dependent antibacterial activity reported for curcumin in the literature [56,57]. In contrast, when incorporated into the coating, curcumin retained antibacterial activity against both bacterial strains under the adopted surface-contact assay conditions. A possible explanation is that the different experimental configurations adopted for the MIC assay and the surface-contact test may influence the observed antibacterial response. Indeed, the two assays should not be interpreted as directly comparable measures of antibacterial activity. However, additional studies would be required to clarify the mechanisms responsible for the different antibacterial responses observed in the two experimental systems.

### 3.2. Chemical and Mechanical Characterization of Produced Coatings

Since viable bacterial recovery from all coatings containing curcumin or Ag^+^ was below the quantification range of the assay against both *E. coli* and *S. aureus*, no concentration-dependent differences in antibacterial performance were observed under the adopted experimental conditions. Therefore, the subsequent structural and mechanical characterization was limited to the coatings containing the highest additive concentration (1 wt%). These formulations were selected because they represent the maximum additive loading and thus the most demanding condition for the coating matrix, allowing the potential influence of the additives on coating structure and mechanical properties, including adhesion and hardness, to be assessed. The visual appearance of the coatings on PC substrates is shown in Figure 1, whereas the corresponding coatings deposited on PMMA substrates were previously reported in our earlier work [45]. No appreciable differences in the visual appearance of the coatings were observed between the two substrate types.

**Figure 3:**
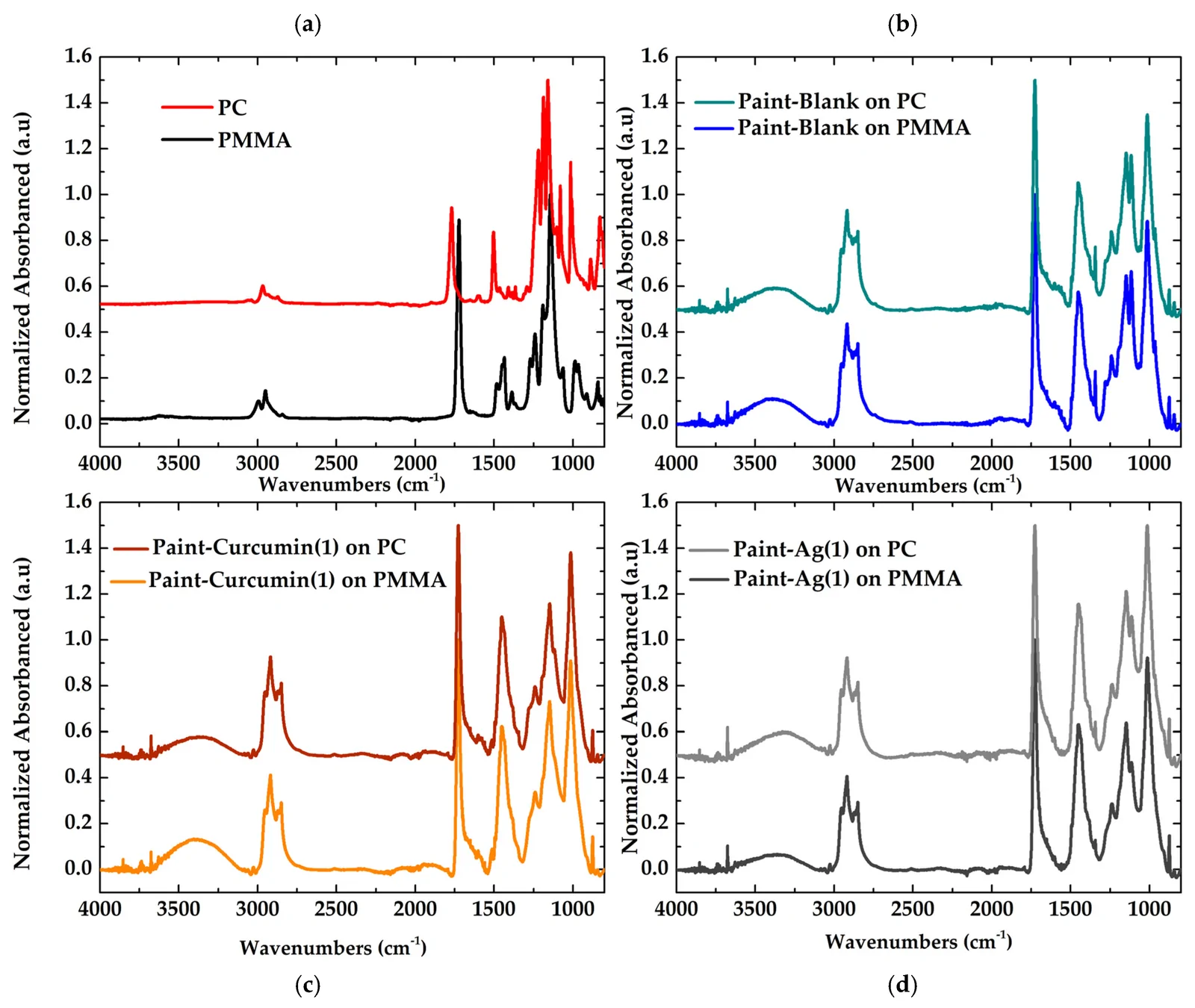
The ATR-FTIR spectra of (**a**) PMMA and PC substrates; (**b**) Paint-Blank on PMMA and PC; (**c**) Paint-Curcumin(1) on PMMA and PC; (**d**) Paint-Ag(1) on PMMA and PC.

To investigate the chemical characteristics of the developed coatings, FT-IR spectra were directly acquired on the coated substrates. To assess the possible contribution of the substrate to the recorded spectra, the FT-IR spectra of the uncoated substrates were also collected and reported for comparison. The resulting spectra are shown in Figure 3.

As shown in Figure 3a, the PMMA and PC substrates exhibit their characteristic FT-IR spectra. In particular, the spectrum of PMMA (Figure 3a, black line) displays a characteristic absorption band at approximately 1722 cm^−1^, corresponding to the stretching vibration of the carbonyl (C=O) group [70,71]. The bands at 1435 and 1142 cm^−1^ are assigned to CH_2_ bending and C–O stretching vibrations, respectively, while the peaks at 966, 840, and 750 cm^−1^ are attributed to C–C stretching and CH_2_ vibrational modes [70,71].

The FT-IR spectrum of the PC substrate (Figure 3a, red line) is characterized by a prominent absorption band at approximately 1770 cm^−1^, attributed to the carbonyl stretching vibration [72]. The band at 1504 cm^−1^ is associated with the C=C stretching vibration of the aromatic rings [72,73]. The peaks at 1219, 1158, 1079, and 1011 cm^−1^ arise from several vibrational modes, including C–O–C stretching, C–C–C bending, and O–C–O stretching vibration [72,73,74]. The band at 827 cm^−1^ is assigned to the out-of-plane C–H deformation of the substituted aromatic rings characteristic of polycarbonate [72,74].

Regarding the produced coatings, no significant differences were observed between the FT-IR spectra of the Paint-Blank (Figure 3b), Paint-Curcumin(1) (Figure 3c) and Paint-Ag(1) (Figure 3d). Since the commercial paint constitutes the major component of the formulation, the recorded spectra are dominated by the characteristic absorption bands of the paint matrix.

The commercial paint is described as acrylic resin-based in publicly available product documentation.. Consistent with this description, the FTIR spectrum exhibits characteristic bands associated with acrylic resins [75,76]. In particular, the broad band at approximately 3400 cm^−1^ can be attributed to O–H stretching vibrations, while the bands at 2919 and 2850 cm^−1^ can be assigned to the stretching vibrations of C–H. The absorption band at 1725 cm^−1^ is likely related to the stretching vibration of the carbonyl (C=O) group [75,76,77]. The bands at 1452, 1241, 1147, and 1011 cm^−1^ can be attributed to CH_2_ bending and C–O and C–C stretching vibrations [75,76,77].

No appreciable differences are observed between the FT-IR spectra of the coatings deposited on PC and those deposited on PMMA. This finding can probably be attributed to the relatively high coating thickness (>30 μm), which may limit the contribution of the underlying substrates to the recorded spectra.

In addition, no distinct new absorption bands attributable to the incorporated curcumin or Ag^+^ additive were observed. Only slight changes in the shape of the bands in the 1147–1012 cm^−1^ region were observed. The absence of distinct absorption bands attributable to the incorporated additives can be explained by their relatively low concentration (1 wt%) compared with the acrylic paint matrix. In particular, the intense absorption bands arising from the paint matrix may overlap or mask the spectral features associated with the additive.

Pencil hardness (ASTM D3363) and adhesion (ASTM D3359) tests were conducted to evaluate the mechanical properties of the produced coatings. The results are reported in Table 4.

As shown in Table 4, the Paint-Blank, Paint-Curcumin(1) and Paint-Ag(1) coatings display a hardness of 4B on PC and 4B/5B on PMMA substrates. The pencil hardness values were assigned based on the hardest pencil that did not leave a visible permanent mark on the coating surface [78]. Under the tested conditions, the 3B pencil removed the coating, as shown in Figure S2, whereas the 4B pencil did not produce a mark; accordingly, the coating was classified as 4B. The incorporation of Ag^+^ or curcumin did not substantially affect the hardness, with the coatings remaining classified as 4B on PC and 4B/5B on PMMA.

Several factors can influence the pencil hardness of a coating, including the formulation composition, substrate characteristics, coating thickness, and film formation conditions [79]. Therefore, the relatively low pencil hardness observed in this study cannot be unambiguously attributed to a single factor. Nevertheless, the coating methodology and dilution conditions adopted may have contributed to the measured values. In particular, the addition of approximately 10 wt% distilled water may have affected the amount of material deposited and, consequently, the final coating thickness.

As reported in Table 4, the Paint-Blank coating achieved an adhesion rating of 4B on both PC and PMMA substrates according to ASTM D3359. This classification indicates good coating adhesion, with less than 5% coating removal after the cross-cut tape test. The good adhesion observed may be attributed to effective substrate wetting during spray deposition and favorable interactions between the acrylic resin matrix and the polar surfaces of the PC and PMMA substrates.

**Figure 4:**
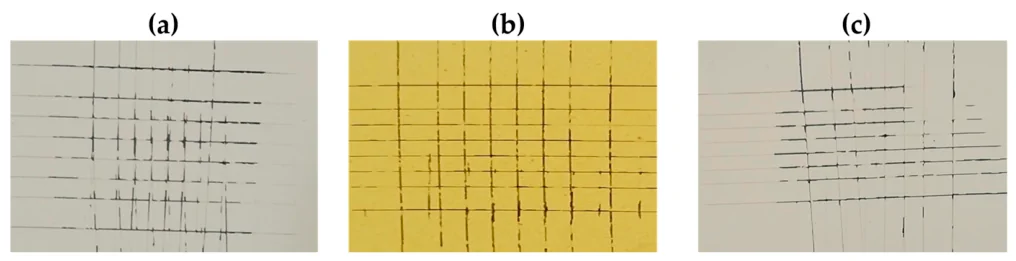
The results of tape test adhesion after adhesive removal for the coatings produced on PC substrates; (**a**) Paint-Blank on PC; (**b**) Paint-Curcumin(1) on PC; (**c**) Paint-Ag(1) on PC. The results on PMMA substrates were reported in SI (Figure S3).

The adhesion profile of the Paint-Curcumin(1) and Paint-Ag(1) coatings is comparable to that of the Paint-Blank and is likewise classified as 4B. These results indicate that incorporating additives into the paint formulation does not substantially alter the adhesion properties of the coatings on either PC or PMMA substrates. The results of tape test adhesion for coatings on PC substrates are shown in Figure 4.

Based on the results of the adhesion and hardness tests, the incorporation of curcumin and Ag^+^ at the investigated concentrations did not substantially affect the mechanical response of the coatings compared with the Paint-Blank formulation.

A key characteristic of antimicrobial coatings is their durability when exposed to water. Many surfaces, such as operating room countertops, door handles, and similar high-contact areas, are routinely cleaned and frequently exposed to moisture. To assess the coatings’ durability under moisture exposure, the samples were subjected to continuous water immersion for a duration of five days.

**Figure 5:**
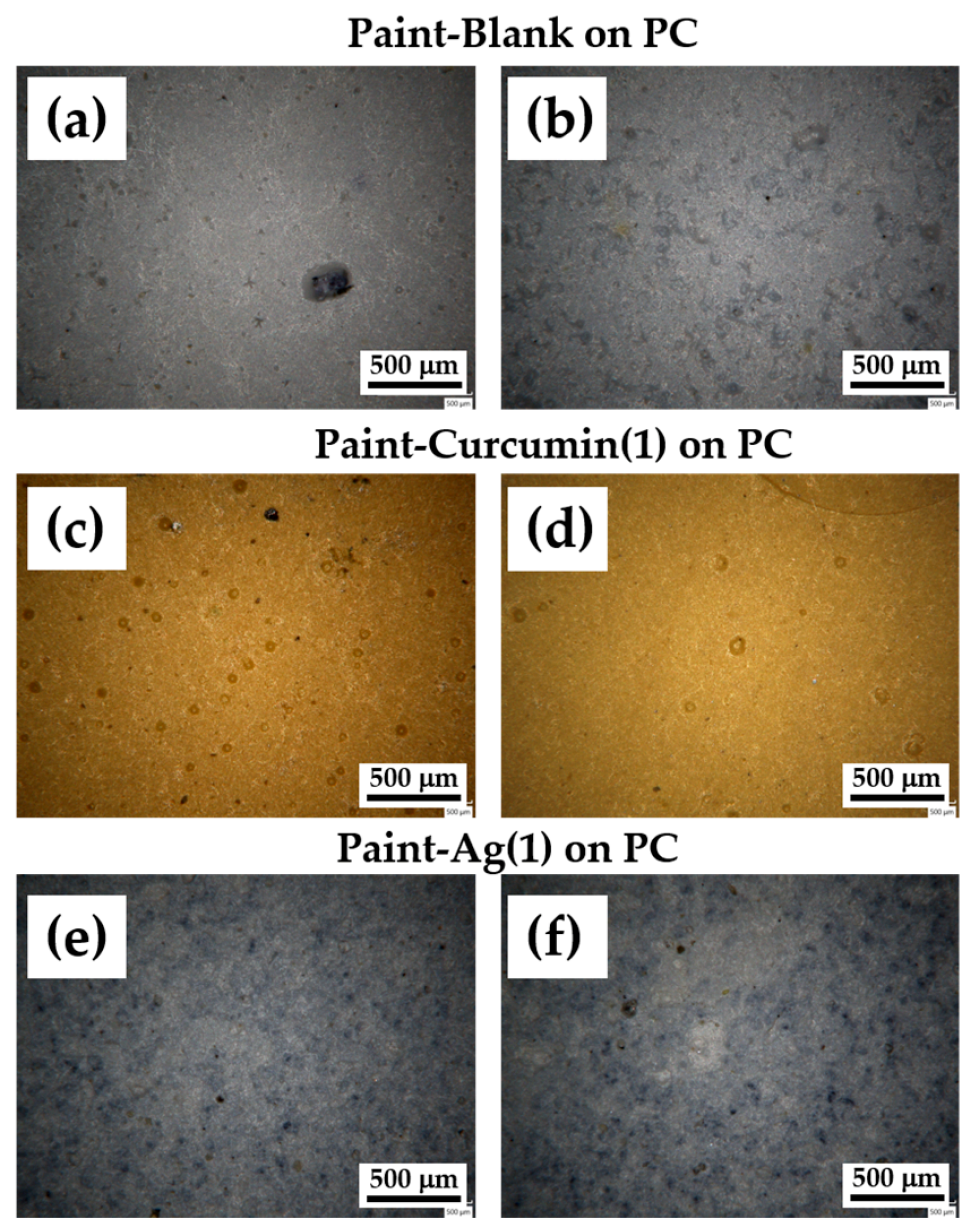
The images of the coatings (**a**) non-immersed area of Paint-Blank; (**b**) water immersed area (5 days) of Paint-Blank; (**c**) non-immersed area of Paint-Curcumin(1); (**d**) water immersed area (5 days) of Paint-Curcumin(1); (**e**) non-immersed area of Paint-Ag(1) and (**f**) water-immersed area of Paint-Ag(1). The images were acquired at 130× magnification after the immersed areas were completely dried. Only the coatings deposited on PC substrates are shown.

Figure 5 shows digital microscope images acquired from two distinct regions of the coated substrates: one region that was immersed in water for five days and a second, non-immersed region used as a reference. Figure 5 shows only the coatings deposited on PC substrates, as those prepared on PMMA exhibited essentially identical behavior. Representative images of the PMMA coatings are included in the Supporting Information (Figure S4).

Based on the images reported in Figure 5, no appreciable differences are observed between the regions immersed in water and the corresponding non-immersed regions. Specifically, after five days of immersion, no visible signs of coating cracking, delamination, or removal were observed, suggesting that the coatings remained macroscopically intact under the investigated conditions.

For the Paint-Curcumin(1) sample, both the immersed and non-immersed regions exhibit a homogeneous orange appearance. No visible discoloration was observed after water immersion. However, the absence of visible discoloration does not exclude possible release or redistribution of curcumin within or from the coating.

Overall, the results of the structural characterization, adhesion, hardness, and water stability tests indicate that the incorporation of curcumin and Ag^+^ did not appreciably affect the investigated properties of the resulting coatings compared with those of the Paint-Blank sample.

The obtained results suggest that the incorporation of curcumin and Ag^+^ at 1wt% did not appreciably affect the adhesion, pencil hardness, or water resistance of the coatings compared with the Paint-Blank reference.

However, the incorporation of curcumin and Ag^+^ reduced bacterial growth. This result indicates the potential of these additives to impart antibacterial activity to the coating systems. Overall, these findings support the feasibility of incorporating curcumin into a commercially available water-based paint to develop coatings with antibacterial activity against both *S. aureus* and *E. coli*.

Nevertheless, some limitations of the present study should be acknowledged.

Although no visible degradation of the coatings was observed after five days of continuous immersion in water, the release of curcumin and Ag^+^ into the aqueous medium was not evaluated. Moreover, the retention of antibacterial activity following water exposure was not investigated. These aspects should be addressed in future studies to better assess the long-term performance and practical applicability of the developed coatings.

The results support the potential use of curcumin in the development of antibacterial coatings and suggest its potential as an alternative to conventional antimicrobial additives. Its natural origin makes it an interesting candidate for the development of more sustainable surface coatings. However, whether the use of curcumin results in a reduced environmental impact cannot be established from the present study.

An additional practical consideration concerns the coloration imparted by curcumin. In the present study, its incorporation resulted in pronounced yellow-to-orange coloration. This characteristic may limit applications in which preservation of the original color of the substrate or paint is required and should therefore be considered in future formulation optimization.

Further evaluations of coating durability, environmental stability, long-term antibacterial activity and coating toxicity are necessary to assess the practical applicability of the developed coatings.

## 4. Conclusions

This work reports the development of antimicrobial coatings based on a commercially available water-based paint containing curcumin, a naturally derived bioactive compound. The antimicrobial efficacy and mechanical properties of the resulting coatings were compared with those of coatings containing an Ag^+^-based additive, a well-established antibacterial agent. The formulations were successfully applied onto PC and PMMA substrates, producing uniform coatings with good adhesion properties but limited pencil hardness. Under the selected surface-contact assay conditions, all Paint-Curcumin and Paint-Ag coatings markedly reduced viable bacterial recovery against both *S. aureus* and *E. coli*, with no colonies recovered within the dilution range used for comparison with the Paint-Blank control.

Moreover, after continuous immersion in water for 5 days, the coatings showed no visible signs of delamination, cracking, or significant discoloration, indicating good stability under the tested conditions.

Overall, this study demonstrates the feasibility of incorporating a natural antimicrobial compound into a commercial paint formulation to produce bio-functional coatings through a simple and scalable spray coating approach.

Several aspects require further investigation, including long-term antibacterial performance, resistance to repeated washing and aging, and the possible release or degradation of curcumin. Toxicity and biocompatibility should also be assessed before considering potential healthcare applications. Further evaluation of its long-term durability and practical applicability will be necessary.

## Figures and Tables

**Table 1:** Experimental conditions and sample names of the antimicrobial paint dispersions.

Dispersions Name	Curcumin wt%	Ag^+^ wt%	Paint Dilution wt%	Substrates
Paint-Blank	-	-	10	PC and PMMA
Paint-Curcumin(0.1)	0.1	-	10	PC
Paint-Curcumin(0.5)	0.5	-	10	PC
Paint-Curcumin(1)	1	-	10	PC and PMMA
Paint-Ag(0.1)	-	0.1	10	PC
Paint-Ag(0.5)	-	0.5	10	PC
Paint-Ag(1)	-	1	10	PC and PMMA

**Table 2:** Determined MIC values for curcumin and Ag^+^ compared with literature values.

Sample	Bacteria Strain	Determined MIC Values	MIC Values (Reference)
Curcumin	*E. coli*	1.25 mg/mL	1.5 mg/mL [56] 2 mg/mL [57]
Curcumin	*S. aureus*	0.15 mg/mL	0.25 mg/mL [56] 0.5 mg/mL [57] 0.2 mg/mL [58]
Ag^+^	*E. coli*	0.62 mg/mL	0.2/0.3 mg/mL [59] Zeolite doped with Ag 0.085 mg/mL [60] Silver nanoparticles tested against *E. coli* U12
Ag^+^	*S. aureus*	0.62 mg/mL	0.625 mg/mL [61] Silver nanoparticles 0.004–0.016 mg/mL [62] Silver-ion nanostructured zeolite

**Table 3:** Antibacterial activity of all coated formulations after 18 h of contact. Values refer to three independent experiments. “No viable colonies recovered” indicates that no colonies were detected at the dilution range used for comparison with the Paint-Blank control. Bacterial reduction was calculated relative to the Paint-Blank control.

Samples	*S. aureus* Viable Recovery	*S. aureus* Bacterial Reduction (%)	*E. coli* Viable Recovery	*E. coli* Bacterial Reduction (%)
Paint-Blank	Countable colonies	-	Countable colonies	-
Paint-Curcumin(0.1)	No viable colonies recovered	100	No viable colonies recovered	100
Paint-Curcumin(0.5)	No viable colonies recovered	100	No viable colonies recovered	100
Paint-Curcumin(1)	No viable colonies recovered	100	No viable colonies recovered	100
Paint-Ag(0.1)	No viable colonies recovered	100	No viable colonies recovered	100
Paint-Ag(0.5)	No viable colonies recovered	100	No viable colonies recovered	100
Paint-Ag(1)	No viable colonies recovered	100	No viable colonies recovered	100

**Table 4:** The results of the pencil hardness and adhesion tests on Paint-Blank, Paint-Curcumin(1), and Paint-Ag(1).

Dispersion	Substrate	Pencil Hardness	Adhesion Test
Paint-Blank	PC	4B	4B
Paint-Curcumin(1)	PC	4B	4B
Paint-Ag(1)	PC	4B	4B
Paint-Blank	PMMA	4B/5B	4B
Paint-Curcumin(1)	PMMA	4B/5B	4B
Paint-Ag(1)	PMMA	4B/5B	4B

## Data Availability

The data supporting the findings of this study are available from the corresponding author upon reasonable request. All relevant experimental details, characterization data, and analysis are included in the manuscript.

## Supplementary Materials

The following supporting information can be downloaded at: https://www.mdpi.com/article/10.3390/biomimetics11090659/s1, Table S1: Coating thickness values for PC substrates; Table S2: Coating thickness values for PMMA substrates; Figure S1: Coating thickness values for the three independent replicates (R1–R3) of each coating formulation. Individual thickness measurements obtained at three different positions on each specimen are shown together with the corresponding mean ± SD. Table S3: Comparison of the antibacterial performance and adhesion properties of the coating developed in this work with those reported in reference studies. Figure S2. Pencil hardness test results for (a) Paint-Blank, (b) Paint-Curcumin(1), and (c) Paint-Ag(1) coatings deposited on PC substrates. Equivalent results were obtained for PMMA substrates. Figure S3. Cross-cut tape adhesion test results for (a) Paint-Blank, (b) Paint-Curcumin(1), and (c) Paint-Ag(1) coatings deposited on PMMA substrates. Figure S4. Digital microscope images of the coatings deposited on PMMA substrates: (a) non-immersed area of Paint-Blank; (b) water-immersed area of Paint-Blank after 5 days; (c) non-immersed area of Paint-Curcumin(1); (d) water-immersed area of Paint-Curcumin(1) after 5 days; (e) non-immersed area of Paint-Ag(1); and (f) water-immersed area of Paint-Ag(1) after 5 days. All images were acquired at 130× magnification after the water-immersed specimens had been completely dried.
