# Supplementary material for: Development of Antimicrobial Coatings by Incorporating Curcumin and Silver-Based Additive into a Commercial Water-Based Paint
Source: Biomimetics (Basel). 2026 Sep 12;11(9):659. doi: 10.3390/biomimetics11090659 (PMC13604864; doi:10.3390/biomimetics11090659)
Supplement: Supplementary file 1 [file biomimetics-11-00659-s001.zip › biomimetics-4442105-supplementary.pdf]

Supporting informations: Development of Antimicrobial Coatings by Incorporating Curcumin and Silver-Based Additive into a Commercial Water-Based Paint

Francesca Pescosolido [1,2], Silvia Vesco [3], Felicia Carotenuto [1,4,*], Andrea Ciammaruconi [5], Florigio Lista [5], Roberto Bei [1], Paolo Di Nardo [1,4,*] and Federica Trovalusci [3]

Coating thickness was determined by measuring the substrate thickness before and after coating deposition at three different positions on each specimen. Three independent specimens were analyzed for each coating formulation (replicates R1–R3). Tables S1 and S2 summarize the mean coating thickness ± standard deviation (SD) for each replicate (calculated from the three measurements performed on each specimen) together with the overall mean ± SD calculated across the three independent replicates for coatings deposited on PC and PMMA substrates, respectively.

Table S1: Coating thickness values for PC substrates.

	**R1 (Mean ± SD)**	**R2 (Mean ± SD)**	**R3 (Mean ± SD)**	**Overall Mean (n=3)**
**Paint-Blank**	40 ± 8	31 ± 6	39 ± 6	37 ± 5
**Paint-Curcumin(0.1)**	89 ± 3	66 ± 13	39 ± 10	65 ± 25
**Paint-Curcumin(0.5)**	47 ± 3	48 ± 15	59 ± 18	51 ± 7
**Paint-Curcumin(1)**	47 ± 5	58 ± 11	89 ± 19	65 ± 22
**Paint-Ag(0.1)**	36 ± 5	51 ± 15	43 ± 6	44 ± 8
**Paint-Ag(0.5)**	66 ± 11	48 ± 7	45 ± 4	53 ± 12
**Paint-Ag(1)**	50 ± 4	71 ± 31	58± 9	60 ± 11

Table S2: Coating thickness values for PMMA substrates.

	**R1 (Mean ± SD)**	**R2 (Mean ± SD)**	**R3 (Mean ± SD)**	**Overall Mean (n=3)**
**Paint-Blank**	22 ± 13	22 ± 7	35 ± 1	26 ± 8
**Paint-Curcumin(1)**	54 ± 11	79 ± 17	66 ± 8	66 ± 12
**Paint-Ag(1)**	46 ± 9	46 ± 4	47 ± 9	46 ± 1

The coating thickness data are also presented as histograms in Figure S1, which show the individual thickness measurements obtained at the three different positions on each specimen, together with the corresponding mean ± SD for each replicate. The relatively large variation in coating thickness (Figure S1) is presumably associated with the manual spray-coating process, which was performed without a mechanical system to control the spraying angle and deposition distance. Despite these variations, all coatings provided complete and uniform coverage of the substrate surface.

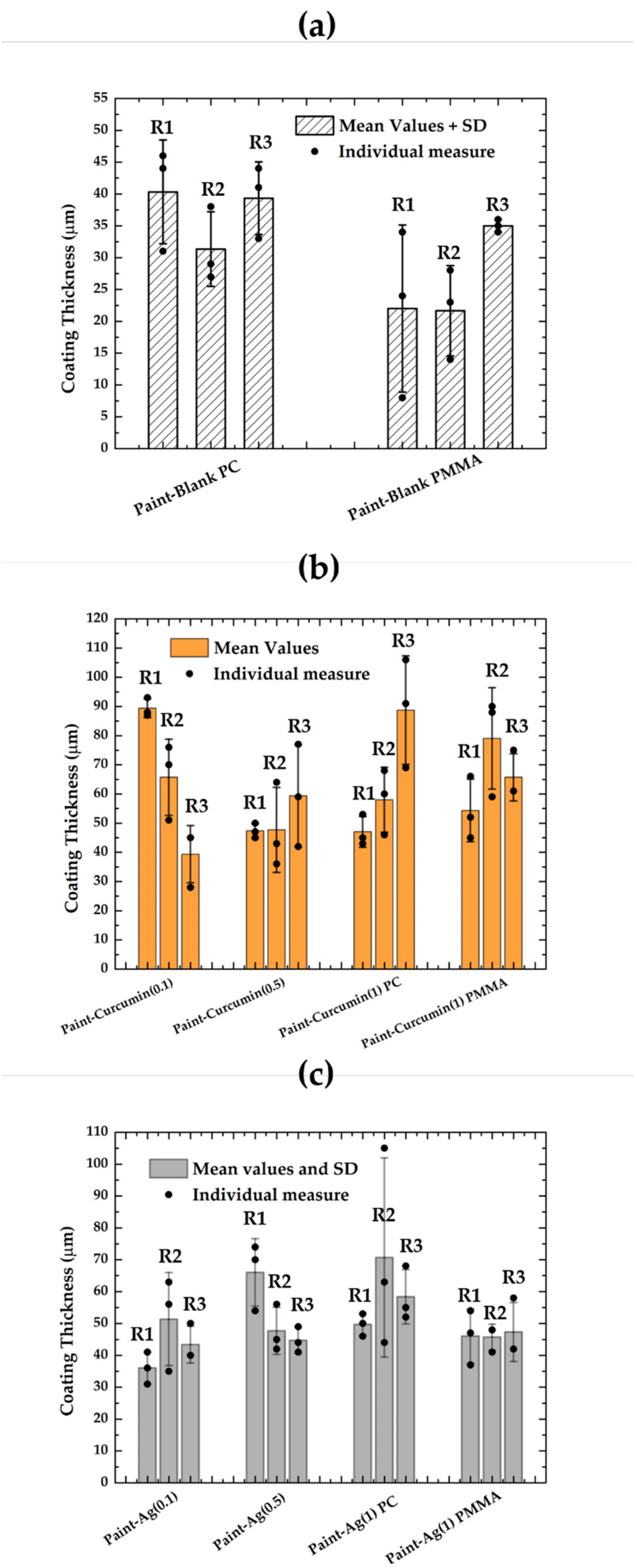

Figure S1: Coating thickness values for the three independent replicates (R1–R3) of each coating formulation. Individual thickness measurements obtained at three different positions on each specimen are shown together with the corresponding mean ± SD.

Table S3 reports a comparison of selected antimicrobial coatings from the literature, including antibacterial performance and, where available, adhesion properties. A direct comparison is not straightforward due to differences in substrates, coating procedures, and antibacterial assessment methods. Nevertheless, the results indicate that the coatings developed in the present work exhibit properties comparable to those reported in the literature.

Table S3: Comparison between the antibacterial assessment and adhesion properies of the coating produced in this work.

Reference	Antibacterial additive	Substrate	Antibacterial assessment	Adhesion properties
[1]	Curcumin/chitosan	Mg-based alloy	Method: Disc-diffusion inhibition area<br>*S.aureus*<br>*E.coli*	-
[2]	Curcumin/acrylic commercial paint	Polymeric coupon	Method: ISO 22196 and JIS Z2801<br>enhanced bacterial reduction, particularly against *S. aureus*	-
[3]	Chitosan/montmor-illonite (MMT)	Metal plates	Method: PN-ISO 22196<br>*E.Coli* ~100%<br>*S. aureus* ~99.98%<br>Bacterial reduction repeated after water immersion (72h)	Adhesion evaluation<br>Method ISO 2409<br>0-1<br>(equivalent to 5B–4B, ASTM D3359)
[4]	Silver-based additive ($AgNO_3$)	Steel panels	Method: ISO 22196<br>~ completely reduction<br>*E. Coli*<br>*S.aureas*<br>Bacterial reduction repeated after degradation test	-
[5]	Silver-zeolite composites	Stainless steel	Method: agar well diffusion zone inhibition<br>Bacterial reduction	Adhesion evaluation<br>Method ASTMD3359<br>4B/5B/3B depending on the silver-zeolite contents
[6]	silver nanocluster silica composite		Method: Inhibition halo test<br>*E.Coli*<br>*S.aureus*<br>Bacterial Reduction	Adhesion ASTMD3359<br>5B
[7]	Chitosan/Citric Acid/Curcumin	Chitosan-based membrane<br>Biodegradable film	Modified ISO 22196 standard<br>*E.Coli*<br>*S.aureus*<br>~ 99.99% to 100%	-
Present work	Curcumin/Acrylic water-based paint<br><br>Silver based additive/Acrylic water-based paint	PC<br>PMMA	Antibacterial activity evaluated using a surface contact assay adapted from ISO 22196.<br>*E.Coli*<br>*S.aureus*<br>~ 100%	Adhesion ASTMD3359<br>4B

The results of the pencil hardness test, performed according to ASTM D3363, are presented in Figure S2. As shown, the 3B pencil completely removed the coating from the PC substrates. Equivalent results were obtained for the PMMA substrates.

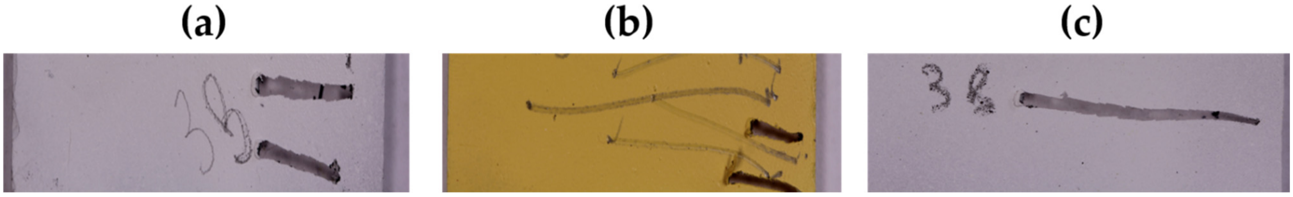

Figure S2. Pencil hardness test results for (a) Paint-Blank, (b) Paint-Curcumin (1), and (c) Paint-Ag (1) coatings deposited on PC substrates. Equivalent results were obtained for PMMA substrates.

The results of the cross-cut tape adhesion test, performed according to ASTM D3359, are shown in Figure S3 for coatings deposited on PMMA substrates.

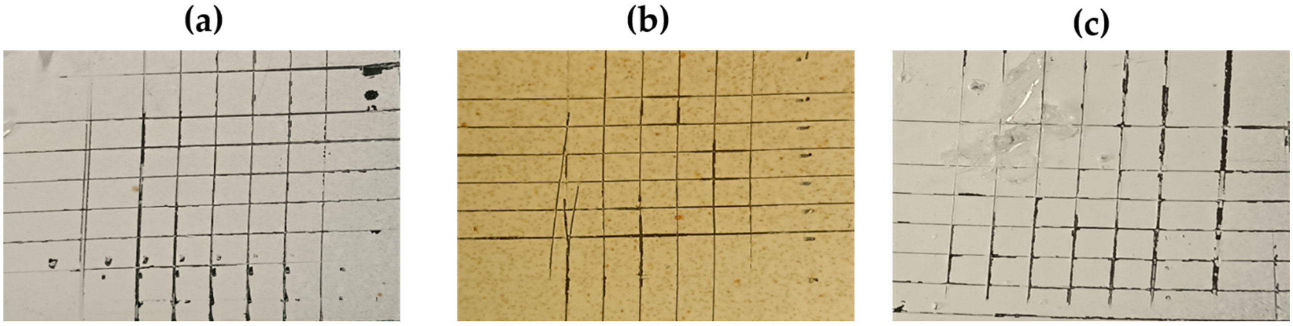

Figure S3. Cross-cut tape adhesion test results for (a) Paint-Blank, (b) Paint-Curcumin (1), and (c) Paint-Ag (1) coatings deposited on PMMA substrates.

Figure S4 shows digital microscope images of the coatings on PMMA substrates after the water immersion test.

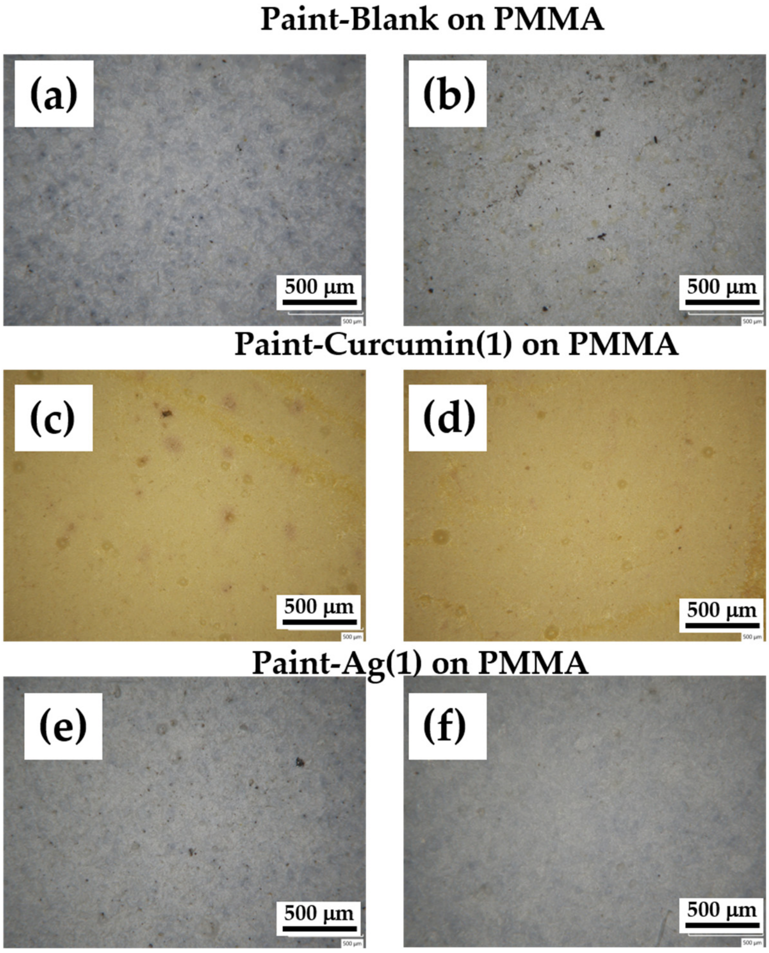

Figure S4. Digital microscope images of the coatings deposited on PMMA substrates: (a) non-immersed area of Paint-Blank; (b) water-immersed area of Paint-Blank after 5 days; (c) non-immersed area of Paint-Curcumin (1); (d) water-immersed area of Paint-Curcumin (1) after 5 days; (e) non-immersed area of Paint-Ag (1); and (f) water-immersed area of Paint-Ag (1) after 5 days. All images were acquired at 130× magnification after the water-immersed specimens had been completely dried.

References

1. Ibrahim, A.A.; Nasr-Esfahani, M.; Bakhsheshi-Rad, H.R. Curcumin-Loaded Chitosan Coatings for Corrosion Protection and Antibacterial Enhancement of HF-Treated Mg Alloy. *JOM* **2025**, *77*, 7962–7974, doi:10.1007/s11837-025-07539-0.
2. Querido, M.M.; Paulo, I.; Hariharakrishnan, S.; Rocha, D.; Barbosa, N.; Galhano dos Santos, R.; Bordado, J.M.; Teixeira, J.P.; Pereira, C.C. Self-Disinfecting Paints with the Natural Antimicrobial Substances: Colophony and Curcumin. *Antibiotics* **2021**, *10*, 1351, doi:10.3390/antibiotics10111351.
3. Pilch-Pitera, B.; Krawczyk, K.; Kędzierski, M.; Pojnar, K.; Lehmann, H.; Czachor-Jadacka, D.; Bieniek, K.; Hilt, M. Antimicrobial Powder Coatings Based on Environmentally Friendly Biocides. *ACS Sustain. Chem. Eng.* **2024**, *12*, 10325–10339, doi:10.1021/acssuschemeng.3c07721.
4. Russo, F.; Furlan, B.; Calovi, M.; Massidda, O.; Rossi, S. Silver-Based Vitreous Enamel Coatings: Assessment of

Their Antimicrobial Activity towards Escherichia Coli and Staphylococcus Aureus before and after Surface Degradation. *Surf. Coatings Technol.* **2022**, *445*, 128702, doi:10.1016/j.surfcoat.2022.128702.

5. Shirani, S.; Emadi, R.; Eslami, A.; Saboori, A. Polyethylene-Silver-Zeolite Composite Coatings on Stainless Steel; Corrosion Resistance and Antimicrobial Characteristics. *Mater. Today Commun.* **2025**, *49*, 113874, doi:10.1016/j.mtcomm.2025.113874.
6. Balagna, C.; Perero, S.; Ferraris, S.; Miola, M.; Fucale, G.; Manfredotti, C.; Battiato, A.; Santella, D.; Vernè, E.; Vittone, E.; et al. Antibacterial Coating on Polymer for Space Application. *Mater. Chem. Phys.* **2012**, *135*, 714–722, doi:10.1016/j.matchemphys.2012.05.049.
7. Cappello, M.; Trouki, C.; Barbani, N.; Lopez, M.J.; Toribio-Gallardo, A.J.; Cristallini, C.; Malik, N.; Rossi, D.; Seggiani, M.; Cinelli, P. Citric Acid-Crosslinked Chitosan Coatings Incorporating Curcumin for Enhancement of Antioxidant and Antimicrobial Properties. *J. Vinyl Addit. Technol.* **2026**, 1–20, doi:10.1002/vnl.70133.
